# Reply to: Characterizing coral skeleton mineralogy with Raman spectroscopy

**DOI:** 10.1038/s41467-018-07602-2

**Published:** 2018-12-14

**Authors:** Anat Akiva, Maayan Neder, Keren Kahil, Rotem Gavriel, Iddo Pinkas, Gil Goobes, Tali Mass

**Affiliations:** 1 Department of Structural Biology, Weizmann Institute of Science, 76100 Rehovot, Israel; 2 Department of Marine Biology, The Leon H. Charney School of Marine Sciences, University of Haifa, Mt. Carmel, 3498838 Haifa, Israel; 3 The Interuniversity Institute of Marine Science, 88103 Eilat, Israel; 4 Department of Chemistry, Bar-Ilan University, 5290002 Ramat Gan, Israel; 5 Department of Chemical Research Support, Weizmann Institute of Science, 76100 Rehovot, Israel; 6 Present Address: Laboratory of Materials and Interface Chemistry and Center for Multiscale Electron Microscopy, Department of Chemical Engineering and Chemistry and Institute for Complex Molecular Systems, Eindhoven University of Technology, 5600 MB Eindhoven, The Netherlands

**Replying to** T. M. DeCarlo *Nature Communications* 10.1038/s41467-018-07601-3 (2018)

The interpretation of mineral phases in the manuscript by Akiva et al. was done by integrating several spectral and imaging methods in a multidisciplinary project. Solid-state NMR, cryo-EM, cryo-EDS, and in-vivo micro-Raman spectroscopy were used. The key point was to report and discuss the observations attained by these techniques, mainly that in contrary to previous assumptions, pre-settled coral larva are not only soft tissue, but also contain mineral particles. Identification of the mineral phases was not the only goal, as we point to the biological control of the mineral skeleton formation by modulation of acidic proteins, generating a different local environment for the deposition of each mineral phase. This is an important step toward understanding how corals build their skeleton. The basic mechanism responsible for the precipitation of the aragonite skeleton in corals has been an enigma for decades, and this study presents another piece in the puzzle. Our current publication supports the observation by photoemission electron microscopy (PEEM), that coral skeletons grow by the aggregation of amorphous calcium carbonate (ACC) particles inside the coral tissue^1^. In the current study, we have cross checked the evidence by ssNMR, micro-Raman, and cryo-EM, showing that coral planulae (free-swimming coral larvae) and polyps of juvenile corals precipitate disordered calcium carbonate particles.

The main findings of this paper “Minerals in the pre-settled coral *Stylophora pistillata* crystallize via protein and ion changes” are: the formation of a mineral phase in an earlier stage than has been reported; the involvement of different mineral phases in the formation of the mature aragonitic skeleton; the involvement of different proteins, represented by the distribution of specific amino acids in the different mineral phases during the coral development, and the correlation between the gene expression of these proteins and specific mineral phases; Finally, the involvement of magnesium ions in the early stages of mineral formation^2^. This paper has a major contribution towards understanding the process of how corals build their initial skeleton.

Many marine organisms produce amorphous calcium carbonate (ACC) as a temporary storage of CaCO_3_ to be transported and used elsewhere within the organism, despite the fact that it is highly unstable in its pure form under physiological conditions.^3^ It is indeed difficult to detect and characterize ACC, and some of the advantages of Raman spectroscopy, are the fact that line positions and linewidths can help us characterize the chemical and physical environment in which the sample is located and material characteristics such as density, order/disorder, temperature and lattice strain.

We appreciate DeCarlo’s concern with the interpretation of the Raman data. Whilst we and others in the community recognize this mineral as disordered calcium carbonate others in the community would identify it as calcite as this is an area of intense disagreement. Whether the mineral is ACC or disordered calcite or both, one of the key findings of our work, that minerals have been identified in the pre-settled larval stage of coral, still stands firm.

However in Akiva et al.^2^ we based our conclusions not only on Raman, but in addition, on Solid-State NMR which is a well-established tool for analyzing mineral phases within biological samples, and as such, the finding that we have located ACC in the juvenile corals cannot be disputed. The same applies to the PEEM data that we compare against, from a previous study^1^. Another support for the characterization of the mineral phase comes from the cryo-SEM combined with EDS that was used showing Mg-containing granules in the size range of 10–100 nm. Similar CaCO_3_ particles, from biogenic and synthetic origin, were previously associated with ACC^4^.

Moreover, using the NMR characterization of the whole organism, we were able to indicate that aragonite and disordered calcium carbonate are the major mineral components in the juvenile coral. A direct excitation experiment of all carbons (^13^C nuclei) in the coral, produced evidence for aragonite which presents a typical narrow peak at 171 whereas an indirect excitation of carbons experiment (cross polarization from protons) produced evidence for ACC which presents a standard broad peak at 169.3 ppm^5,6^. These two prominent spectral features are shown respectively in spectra C and E in figure 4 of our paper. It is noteworthy that a calcite peak, typically observed at 168 ppm in the direct excitation spectrum, is clearly absent from the spectrum of the coral. This has led us to focus on reporting the major components discernable from the spectra of the NMR which cover the entire sample. In the Raman spectra, the standard means of identification of ACC through the absence of lattice bands and through the broadening of the band characteristic of the symmetric stretch of the carbonate ion (the latter not being unique), admittedly made it more challenging to reach conclusive assignment of peaks to ACC. Especially, due to the presence of several mineral phases with varying levels of disorder. Nevertheless, the broad base band observed in the Raman spectra along with a broadened lattice peak at 150–300 cm^−1^ was attributed to a highly disordered mineral phase that was closely matching an ACC phase (clearly observed in the NMR). It may be that this lattice band can raise questions to the extent of order/disorder in the phase and DeCarlo justifiably questions the attribution to a completely amorphic phase of this phase. Yet, the issue is that reporting it as any other phase than ACC would be inconsistent with observations by other techniques reported in the paper.

In summary, the activity of additives (especially biomolecules) and their ability to influence the picture coming out of classical crystallization theory is still an unchartered terrain and requires steady, well-supported experimental evidence, remembering the limitations of each technique. It should be noted that the direct excitation ^13^C NMR measurements were recorded using short flip pulses and a 4-min-long delay between acquisitions to account for the long relaxation times of crystalline calcium carbonate phases (in the order of 10 s of minutes). This approach to expedite signal acquisition was tuned on biomimetically prepared calcite and proven successful, however, in samples where calcite is a minor phase, it is possible that the ^13^C signal from this mineral phase to be undetectable due to this technical limitation.

DeCarlo states that aragonite cannot evolve out of calcite directly and there is evidence that this claim is thermodynamically sound in a pure calcium carbonate binary system. However, this is not the situation in biogenic minerals, such as a coral skeleton which contain numerous additives. The presence of Mg^2+^ ions and biomolecules will utterly disrupt the energy balance and crystallization pathways. Magnesium can dope calcite crystals forming Mg-calcite that is thermodynamically very different than calcite, making transformations to aragonite realistic by processes such as dissolution-precipitation, as DeCarlo is well aware of. We have not claimed that calcite is turning into aragonite. The perception that minerals in coral are formed only by thermodynamic considerations neglects the role that biology plays within the formation of the coral skeleton. In addition to this, our spectra that DeCarlo claims to show a phase similar to calcite in coral larvae are much like other published spectra assigned as phases containing ACC: Figure 3A in Gilis et al.^1^ presents a spectrum from the coral *P. damicornis*, and figure 4 in Wehrmeister et al.^2^, shows spectra of ACC in *P. scaber* (a crustacean) and in *P. maxima* (a bivalve). We are dealing with nascent material out of which the ACC component is known to be of nano-metric dimensions while the disordered crystalline phase is of larger dimension making it much harder to distinguish in the Raman spectrum. We agree that the Raman spectra suggested the presence of disordered calcium carbonate, most likely disordered calcite prior to settlement. The latter might indicate that calcite may play an important transient role during in biogenic CaCO_3_ maturation.

The utilization of ACC is quite common throughout the tree of life, so common that there is a variation on this theme by the use of high magnesium calcium carbonate, as has been shown in many organisms^3^. Additionally, magnesium calcium carbonate has been shown in vitro to occur via a transient ACC phase^4,5^. The use of magnesium in calcium carbonate is seen both in cases where the end product is aragonite as in coral^1^, but also by organisms producing calcite^6^. It is easy to overlook the presence of ACC in biological tissue, unless it is unusually stable. It is also difficult to detect the presence of ACC when it occurs together with one of the crystalline polymorphs, as signal of the latter is much stronger.

The issue of how disordered or amorphous a material is and at what stage can it be defined as a crystalline material is a very important and an extremely debatable intriguing question. In fact, materials that will have features that can resemble one phase or another in spectroscopic methods such as Raman, FTIR, and NMR may still appear amorphous in x-ray and electron diffraction due to the wave diffraction limit. Defining any form of non-crystalline phase based on only one spectroscopic method can be misleading and misinterpreted.

Akiva et al.^7^ describe new findings from the study of juvenile corals. These findings characterize mineral phases by several techniques, as well as correlate the existence of these mineral phases with proteins activated by the coral. There is strong evidence of the presence of ACC in the juvenile corals with or without the micro-Raman data. This work goes a long way towards explaining how corals precipitate calcium carbonate skeletons and clearly shows that the process is biologically controlled.
